# The experiences of families of children with cerebral palsy and complex disability after three years accessing the National Disability Insurance Scheme

**DOI:** 10.1111/1440-1630.12973

**Published:** 2024-06-05

**Authors:** Maddison O'Neill, Helen Bourke‐Taylor, Anoo Bhopti, Claire Cotter

**Affiliations:** ^1^ Department of Occupational Therapy, School of Primary and Allied Health Care, Faculty of Medicine Nursing and Health Sciences Monash University Frankston VIC Australia; ^2^ Cerebral Palsy Education Centre Glen Waverley VIC Australia

**Keywords:** childhood disability, families, National Disability Insurance Scheme (NDIS), parents, qualitative research

## Abstract

**Introduction:**

In Australia, children with cerebral palsy and complex disability receive funded supports through the National Disability Insurance Scheme (NDIS). This individualised funding scheme requires parents to navigate and advocate on behalf of their child, supported by expert reports, recommendations, and allied health services. Supports aim to enable participation in all areas of daily life, which may be otherwise largely inaccessible to children with complex disability and their families. This study aimed to explore the experiences of families of children with complex disability after 3 years accessing the NDIS.

**Methods:**

A qualitative research design with a demographic questionnaire and in‐depth interview was undertaken. Purposive sampling was used to recruit participants from one organisation providing occupational therapy and other allied health services. Data analysis implemented Braun and Clarke's thematic approach to examine the experiences of participants.

**Consumer and Community Involvement:**

This research was conducted with a registered National Disability Insurance Scheme provider to give voice to parent consumers who raise children with complex disability.

**Findings:**

Seven mothers and one father (N = 8) of children with complex disability were interviewed. Most parents reported increased success and satisfaction navigating the scheme. Five overall themes were generated from the data: *pivotal roles of families*, *parental empowerment*, *life‐changing equipment*, *the fallibility of the scheme*, and *a critical scheme*.

**Conclusion:**

Parents reported reliance on the scheme for their child's basic daily care and a more enriched life for their child and family. Parents were grateful for the scheme but experienced inconsistencies, navigation difficulties, and variable choice and control. Most parents had fears about the sustainability of the scheme, translating into uncertainty about their child's future. Allied health professionals, including occupational therapists, are key advocates for children with complex disability and their families. Collaboration through sharing knowledge and skills to support children, their families, and carers is key to empowering parents to navigate the NDIS.

**PLAIN LANGUAGE SUMMARY:**

The National Disability Insurance Scheme (NDIS) provides funding for people with permanent and significant disability. Children with cerebral palsy (and other complex disability) are lifetime users of the NDIS. For children with complex disability, their families are crucial to ensuring that their daily needs are being met, including providing medication. Previous research indicated that parents rely on the NDIS to support their children; however, there have been various challenges such as long wait times for equipment and difficulty understanding how to use the scheme. This study explored the experiences of families of children with complex disability, after more than 3 years of being an NDIS participant. Eight parents from one therapy service provider completed a short questionnaire about themselves, their child, and their family, followed by an interview with the first author. Four authors (occupational therapists) worked together to design and implement this study. The findings highlighted several key points: the important role of parents as caregivers; parents became more knowledgeable and confident to navigate the NDIS with time; equipment funded by the NDIS was life‐changing; the NDIS has ongoing issues; and the crucial nature of the NDIS. Occupational therapists can be extremely important to families, including with supporting families to navigate the NDIS and advocating for them. Occupational therapists must stay current with their knowledge of the NDIS as they provide lifetime support, including prescribing equipment, technology, and home modifications.

**Key Points for Occupational Therapy**

- After 3 years, families raising children with complex disability continue to find the NDIS to be crucial and beneficial, yet challenging.
- Occupational therapists have a pivotal role in advocating for children with disabilities, empowering parents, and providing research evidence about ways to improve the scheme for participants.
- Understanding the experiences of families of NDIS participants can ensure occupational therapists are providing appropriate equipment and technology, therapy services, and other supports.

## 1 INTRODUCTION

The National Disability Insurance Scheme (NDIS) has reformed the disability sector within Australia for people with permanent and significant disabilities (NDIS, 2023a). The NDIS is a social insurance model, underpinned by the principles of participant choice and control, and reasonable and necessary supports, to help participants work towards life goals (NDIS, 2023a). As of June 2023, 610,502 Australians were registered NDIS participants, with an average support budget of $74,900 (NDIS, 2023b). People with cerebral palsy are substantive users of the NDIS, receiving an average support budget of $130,500 (NDIS, 2023c). There are currently 17,334 NDIS participants with cerebral palsy, with 42% being 18 years and younger (NDIS, 2023c). Children with cerebral palsy are lifetime users of the NDIS (Graham et al., 2016).

Cerebral palsy is a permanent “motor disorder … often accompanied by disturbances of sensation, perception, cognition, communication, and behaviour” (Rosenbaum et al., 2007, p. 9). Thus, cerebral palsy can be considered a complex disability, defined to be inclusive of multiple disabilities and difficulties with an origin and management that is multifaceted (Dan, 2021). Children with complex disability require additional technology and supports and a multidisciplinary allied health team to be active members of their community (Cadwgan et al., 2019). Occupational therapists are major service providers for this population, assessing occupational performance and providing interventions including assistive technology, to enhance participation in everyday activities (Occupational Therapy Australia, 2019).

The families of children with complex disability are instrumental in their daily life, with the co‐occupation of parents influencing the child's overall participation (Bourke‐Taylor et al., 2010). Co‐occupation refers to participation in an occupation that is the result of interactions between two people, including tasks completed separately that influence both people (Pierce, 2009). Ultimately, this profound caregiving can cause difficulties for parents participating in their own routines (Ranger et al., 2021), including productivity and leisure occupations (Bhopti, 2016). Hence, the NDIS is important for parents who provide profound caring that includes managing services, access to funds and services, and a multitude of other direct and indirect care activities (Bourke‐Taylor et al., 2010).

The implementation of the NDIS meant that parents had to learn how to navigate the scheme as it was being rolled out. From inception, researchers and policy experts have iterated the importance of listening to the lived experience of scheme participants to shape the NDIS to accommodate the real‐life needs of Australians (Thill, 2015). In response, the current research team conducted a qualitative study to investigate the experiences of parents of children with cerebral palsy and complex disability, after 1 year within the scheme (Smethurst et al., 2021). In‐depth interviews with eight parents revealed that families relied heavily on equipment to manage everyday life and be able to participate in family activities; that wait times for equipment were prohibitive to safe daily management and participation for their child and family; that navigating the NDIS was frustrating due to lack of knowledge about their child's condition or what their child would require and should receive, with administrative barriers providing reduced choice and control; and a sense of gratefulness and hope, tempered by fear for their child's future, due to the future viability of the NDIS (Smethurst et al., 2021).

Other research with parents and NDIS participants concurred with these findings. Two qualitative studies and one mixed method study also reported parent and participants experiences that the NDIS was difficult to navigate and had a high associated administrative burden (Loadsman & Donelly, 2021; Prowse et al., 2022; Yates et al., 2021). Parents of NDIS participants in two of these studies reported that they felt inadequately educated on how to manage and utilise their child's NDIS plan (Prowse et al., 2022; Russo et al., 2021). Other research reported that it was crucial to have the ability to advocate and actively voice the NDIS participants' needs (Barclay et al., 2020; Barr et al., 2021; Prowse et al., 2022; Russo et al., 2021). Other studies found that some participants felt unsupported by the scheme (Loadsman & Donelly, 2021) and that personnel lacked the skills required to work within the disability sector (Gavidia‐Payne, 2020). Similar to our previous research, other research with parents acknowledged that the supports received through the NDIS allowed for essential assistive technology and therapy services to be accessed (Prowse et al., 2022).

One major event that has occurred since the NDIS was rolled out across Australia is the COVID‐19 pandemic. The influence of the COVID‐19 pandemic on the NDIS experiences of families of children with complex disability requires investigations. Yates et al. (2021) reported that families received less support in the home due to cancelled services and fears of contracting the virus. Thus, telepractice‐delivered services were initiated. Masi et al. (2021) investigated the wellbeing of children with neurodevelopmental disabilities (NDIS participants) and their parents during the pandemic, with 68.8% of the 302 parents surveyed reported to use telepractice. Only 30% of these parents reported satisfaction with using this online form for their child's NDIS services.

This study aims to deepen the understanding of consumer experiences, to ascertain if any changes have occurred for families raising children with cerebral palsy and complex disability, following 3 years in the scheme and to understand the COVID‐19 lockdown impacts on families. Hence, the aim was to explore the experiences of families of children with complex disability, after more than 3 years of accessing the NDIS. Three research questions were posed:

1. What are the experiences of families of children with complex disability who have received NDIS services for three or more years?
2. What opinions do parents of children with complex disability have about the supports they have received through the NDIS to enable participation in everyday activities?
3. How did COVID‐19 impact the experiences of NDIS participant children with complex disability and their families?

## 2 METHODS

### 2.1 Positionality statement

All authors identify as female and are qualified occupational therapists by training. All authors acknowledge the systems and structures that afford us unearned privilege and are committed to improving our understanding and practice around decolonising research, guided by feminist, Indigenist and decolonising perspectives, and by people with lived experiences different than their own.

### 2.2 Study design

This qualitative study explored the experiences of families of children with complex disability, with a pragmatic approach used to answer practical problems (Allemang et al., 2021). This study is also underpinned by the constructivist paradigm, which recognises that each person's established views are influenced by the contexts in which they live (Creswell, 2018). Ethics approval was obtained from Monash University Human Research Ethics Committee (project number 17641).

### 2.3 Research setting

The research setting for this project was the same organisation as the previous study, Cerebral Palsy Education Centre (CPEC), based in Victoria. Specifically serving people with CP and similar neurological conditions from birth to 65 years old, about 250 families currently access regular CPEC services (CPEC, 2022). Services offered include individual and group allied health, NDIS plan support, along with various educational workshops and training sessions (CPEC, 2022). The vision of CPEC is a world in which all people with CP and like conditions can actively participate in life, by being included within their society (CPEC, 2022). Author one attended the centre for 22 days over 6 months to better understand the situation of families attending the centre. Prolonged engagement allowed for participant experiences to be better understood (Fossey et al., 2002). Author four had prolonged, 40 year engagement with the population as an occupational therapist and was key in deriving the interview guide and providing contextual background to NDIS services.

### 2.4 Participants and recruitment

In accordance with the previous study by Smethurst et al. (2021), purposive sampling was used for this single organisation study, with a new set of participants recruited. Over 10 weeks, participants were recruited via flyers displayed in the reception of the service provider, along with blanket email to all the families who access CPEC services. The fourth author worked at CPEC and was shielded to recruitment uptake to maintain confidentiality, prevent coercion, and minimise power relationships influencing participant interviews. Participants were eligible to participate in this study if they were a parent of a child (aged under 18 years); their child had been an NDIS participant for at least 3 years; their child received services from CPEC for at least 1 year in the previous 3 years; and they were the parent most involved in their child's NDIS plan. Parents were excluded if their child had been a hospital inpatient for more than 3 months in the last 12 months. This exclusion criterion was created to ensure parents had recent experiences within the NDIS, rather than prolonged experiences in the health‐care sectors.

### 2.5 Data collection

Participants followed a QR code on flyers and in emails to access the explanatory statement online, provide informed consent and their contact details in the online survey, and answer basic demographic questions. The first author provided the flyers but had no role in recruiting families. After informed consent and participants contact details were received, the first author then contacted eligible participants to schedule an in‐depth interview. Participant confidentiality was maintained at all times to allow participants to express their own feelings, experiences, and thoughts without service providers knowledge (i.e., author 4) of their identity (Serry & Liamputtong, 2017). The devised, piloting and revised interview guide (see Table 1) was used by the first author to complete interviews over the phone, videoconferencing, or face‐to‐face (as per participants' preference). The first author had no prior knowledge of any participant or family. Interviews were recorded and then transcribed verbatim. Authors one and two were involved in data analysis at all stages of the planned analysis (see Table 2). Member checking occurred, through sending the transcripts back to participants, to ensure accuracy (Creswell, 2018). All data were securely stored on password‐protected computers, which was only accessible by the first and second authors.

**TABLE 1:** Interview guide used for participants.

Interview guide
Can you please describe your child's typical daily routine? When your child's NDIS plan has been nearing its reassessment date or you had a plan review, how was this process? If you have received assistive technology or equipment (for example to help with mobility) what was this process? Would you say you have a good understanding of the NDIS processes and how the whole scheme works? How would you describe the knowledge of NDIS planners and those working in the NDIS, about your child's condition and needs? During NDIS planning meetings, did you feel like you were in control and could make your own decisions about your child's required supports? If you were able to provide your thoughts back to the NDIS, what are the main things you would tell them? During COVID‐19 and the lockdowns, did your child's daily needs change? Did your child's NDIS plan change during COVID‐19 and the lockdowns? How would you describe the support you and your child received from the NDIS during the COVID‐19 pandemic and lockdowns? How has your family's life changed by your child being an NDIS participant? Are there any other experiences or opinions in general that you have had involving the NDIS that you would like me to know or be aware of?

**TABLE 2:** Six phases of thematic analysis adapted from Braun and Clarke (2021).

Phase	Description
1 Familiarising yourself with the data	The first author audio‐recorded and transcribed all interviews verbatim. The first author re‐listened to each audio recording after transcribing was complete. The first and second authors read and re‐read all transcripts.
2 Coding	The first and second authors coded all interview transcripts independently. The parts of the data that were identified as interesting, had meaning or relevance to the three research questions, were coded. New codes were developed, along with the use and refinement of existing codes, where relevant. Following independent coding, the first two authors discussed the codes derived from the data and comparisons were made within each interview. This was done to ensure that there was consistency in the codes and a minimisation of researcher bias.
3 Generating initial themes	The first and second authors generated the initial themes from common codes across all interviews. Grouping of the codes occurred through looking for patterns within the data, across interviews. Codes sharing a similar idea were preliminary grouped using a spreadsheet, with consideration of the three research questions. Once these codes were placed into these initial groupings, the first two authors reviewed and discussed the themes. Authors collaborated over initial themes that were generated. Changes to the initial themes were discussed and codes were moved accordingly. This created candidate themes.
4 Developing and reviewing themes	The candidate themes were then compared back to the codes and the full sets of data (transcripts), by the first and second authors. Each theme was analysed against the data to ensure it covered the most important and common meanings within the overall data with authors one, two, and three reaching consensus. Additionally, each theme was compared to the three research questions within this study by authors one, two, and three, to ensure relevance.
5 Refining, defining and naming themes	Each identified theme was refined, through authors one and two writing a definition of each theme. This was done to ensure the themes were clear, unique, and encompassed quality findings into the analysis. Each theme name was devised, with names chosen that were both informative and represented the data. Names were carefully chosen to ensure they provided a clear direction of the analysis, rather than just an overall summary. Authors 1 and 2 reached consensus over themes and definitions.
6 Writing up	The first author wrote the final findings, with contributions from authors two and three. The writing of the findings began during the data analysis and thus, evolved and changed over time with discussions and changes to themes. The final writing of the analysis involved the selection of participant quotes that captured the data well.

### 2.6 Data analysis

The six phases of thematic analysis were used to guide the data analysis process, as outlined by Braun and Clarke (2021). Researcher triangulation occurred throughout the analysis via weekly meetings between authors one and two to ensure the accuracy of the findings (Creswell, 2018). Following transcription, interviews were cleaned and de‐identified, with pseudonyms applied for all study participants. Refer to Table 2 for the data analysis process.

### 2.7 Trustworthiness

Trustworthiness was achieved through applying the principles of making the data collection and analysis process credible, transferable, dependable, and confirmable (Lysack et al., 2017). Authors were reflexive in their practices during data analysis, through collectively discussing the proposed codes and themes using inductive mindset (Braun & Clarke, 2021). This contributed to the credibility of the data. Weekly meetings between authors one and two occurred, with notes and processes recorded accurately, which allowed for opportunities to reflect and reach consensus, ensuring dependability. To ensure transferability, descriptions of the population were accurate and the methodological processes undertaken were included in this paper (Liamputtong, 2017). Data were analysed by the researchers independently before collaborating to ensure the findings were consistent, dependable, and confirmable (Liamputtong, 2017).

## 3 FINDINGS

Seven mothers and one father (N = 8) participated in interviews that ranged from 30 to 80 minutes in length. Interviews were completed over Zoom (n = 6), phone (n = 1), and in‐person (n = 1), based on participants' preferences. Participants' education levels included completion of a diploma (n = 1), certificate/TAFE qualification (n = 1), undergraduate degree (n = 4), and Masters/postgraduate study (n = 2). Participants had a total of four children (n = 1), three children (n = 4), two children (n = 1), or one child (n = 2). Participants in this study worked full‐time (n = 1), part‐time (n = 3), or did not work (n = 4). Refer to Table 3 for further information about each NDIS participant.

**TABLE 3:** Demographic information about the NDIS participants.

Demographic data relating to the child (NDIS participant)								
Parent (pseudonym)	Length of time in NDIS (years)	Child's age (years)	Primary diagnosis	Gross motor function classification system level	NDIS plan‐management	Mobility aids used	Communication device used	NDIS supports: Core supports^a^; capacity building supports^b^; capital supports^c^
Alice	5	6–10	CP	3	Plan‐managed^d^	Yes, manual wheelchair	No	All
Grace	6	6–10	Genetic disorder/like condition to CP	5	Self‐managed^e^	Yes, manual wheelchair	Yes	All
Natalie	4	6–10	CP	5	Self‐managed	Yes, manual wheelchair	Yes	All
Dave	4	0–5	CP	5	Half self‐managed, half plan‐managed	Yes, assistive stroller	No	All
Linda	4	11–15	CP	5	Self‐managed	Yes, powered wheelchair	Yes	All
Maria	5	11–15	CP	5	Half self‐managed, half plan‐managed	Yes, powered wheelchair	No	All
Rachel	3	0–5	CP	4	Plan‐managed	Yes, assistive stroller	Yes	All
Nicole	4	6–10	CP	3	Plan‐managed	Yes, manual wheelchair	Yes	All

^a^ Core supports provide assistance with daily activities and everyday needs.

^b^ Capacity building supports aim to promote independence and develop skills that work towards participants' goals.

^c^ Capital supports allow for specific NDIS determined, one‐off purchases of expensive equipment and/or home/vehicle modifications (NDIS, 2020).

^d^ Plan managed: The plan is managed by a plan manager, who assists with the management and organisation of the NDIS participants' funds. Plan managers can organise supports for participants and pay service providers, instead of participants.

^e^ Self‐managed: The participant or a nominee manages their own plan and NDIS funds, thus choosing the service providers and controlling the finances themselves. *Note*: no families were Agency (NDIA) managed: The National Disability Insurance Agency (NDIA) manages the NDIS plan, in which the NDIA directly controls the financial aspects of receiving NDIS funded services.

**FIGURE 1:**
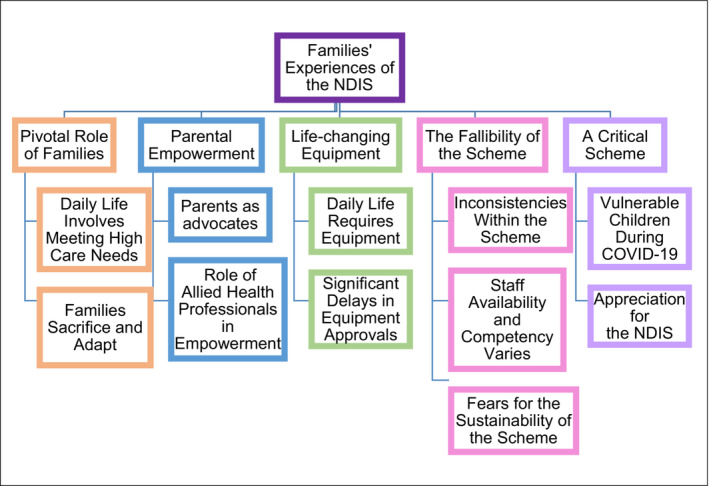
Themes and subthemes derived from the data.

Overall, five themes were derived from the data as displayed in Figure 1. The themes and subthemes with representative quotes are presented below.

### 3.1 Pivotal role of families

Participants described the high level of care and support required for their children to live and the impacts on the wider family. The following subthemes are presented below: *daily life involves meeting high care needs* and *families sacrifice and adapt*.

#### 3.1.1 Daily life involves meeting high care needs

Linda stated their child needed “full support for everything,” a reality shared by all participants. Seven participants explained the importance of medication and medical equipment for their child. Dave described medication to be the “only really kind of consistent thing,” administered “six times a day.” Four participants stated that their child is fed via a percutaneous endoscopic gastrostomy (PEG) tube.

Support workers, often referred to as *carers*, were employed using NDIS funds to assist with daily caregiving within the home and outside in community activities. Linda mentioned that this respite allowed her family to “recharge our batteries,” which was important because “there's a lot of care involved.” Other parents described how support workers enabled their child to attend their own preferred activities while the family engaged with the activities of other children in the family elsewhere. Grace summarised her child's morning routine:

He has feeding overnight, and then in the morning we turn that off, and then he has medications at 6 am and then at about 7 am we have a carer come and gets [child] into the shower, washes his hair, and everything and gives, then gives him breakfast via a tube, he's got a peg.

#### 3.1.2 Families sacrifice and adapt

Seven participants described challenges with retaining employment, due to the high demands of caregiving. Participants described “adapting work commitments” (Alice) around caregiving responsibilities and being so “burnt‐out” (Dave) they had to cease employment.

We have a carer that picks her up on a couple of afternoons a week when I am working, but, yeah, the rest of the time … if she's got hospital appointments or anything like that, it's just my husband and I, we just try to land it on a day off or, or take a, shuffle it around, it's like who's taking the sick leave this time. (Alice)

However, one participant explained that the support of the NDIS allowed them to return to full‐time work, which is “great for my mental health” (Linda).

Participants stated that “we're people who are trying to cope” (Nicole) and “just trying to function” (Dave). Dave identified their need for psychological support while navigating his role as a parent of “a child with a severe disability,” as he described his “whole world to be turned upside down.” Multiple participants disclosed the significant toll that caregiving had on their mental health and wellbeing.

### 3.2 Parental empowerment

It was evident that over years of engagement with the NDIS, parents had a greater understanding of the NDIS and felt more empowered to manage on behalf of their child. Two subthemes emerged: *parents as advocates* and the *role of allied health professionals in empowerment*.

#### 3.2.1 Parents as advocates

Seven parents described advocacy efforts where they had to “fight” (Nicole) for their child's needs. Linda stated that the “first year [in the NDIS] was horrendous,” but their plan is “fantastic at the moment,” as they had “learnt to work the system.” Alice, Grace, and Natalie also developed an understanding of how to navigate the NDIS. In contrast, Maria was the only parent who felt that the NDIA had “totally ignored” evidence from health professionals for supports to benefit her child and family. Parents disclosed the need for the whole family to be considered, with Nicole asking “can it [the NDIS] be family‐centred?”

Two participants agreed that the NDIS provided a good quantity of supports, but the “fight to get it is harder” (Maria). Dave proposed that success within the NDIS comes down to a parent's “ability to wade through Government speak.” The importance of discussing the child's inabilities to attain NDIS funding was also identified.

It's overwhelming to kind of put everything on paper … because you have to kind of talk about all the difficulties you have and all the bad things, I mean that was kind of hard. (Rachel)

Similarly, five parents emphasised their experience, that “navigating the NDIS is stressful and taxing on parents” (Grace).

#### 3.2.2 Role of allied health professionals in empowerment

Alice stated that they “would have drowned” without allied health professional support, during NDIS applications and review processes. Three participants reported that their children's occupational therapists were knowledgeable of the NDIS and they “know what they can ask for” (Alice). Linda identified the knowledge of therapists as crucial, to have a “strong team” around the NDIS participant.

Three participants mentioned that health professionals should have a greater say in a child's entitled NDIS supports, as there “needs to be educated decisions that are being made” by professionals who knew the child (Grace).

### 3.3 Life‐changing equipment

Participants described the child and family's use and reliance on assistive technology, often called *equipment*. The following subthemes emerged: *daily life requires equipment* and *significant delays in equipment approvals*.

#### 3.3.1 Daily life requires equipment

Participants explained how equipment enabled their child to actively participate within the family, to avoid feeling like “everything is a hassle,” which is how families felt without the right equipment for their child (Alice). Without equipment, their children “can't get out of the house” (Grace) or “access the school bus” (Nicole).

#### 3.3.2 Significant delays in equipment approvals

Participants described timelines as “pretty outrageous” (Dave), with delays ranging from months to years. Grace explained that they had “been trying for years to get a hoist” despite their child being “so significantly disabled.” The follow‐up with the NDIA required to track the progress of equipment applications was deemed to be “exhausting” (Maria). Dave explained that they have “built things” to manage the prolonged wait times:

If you factor in your time, energy as the cost, then you're like, well, it doesn't even actually end up benefitting us if NDIS funds it, because we've had to go through all the inconvenience of not having it plus the work to get it. (Dave)

Nicole described how her child “actually fell out of her wheelchair on the bus, because the equipment wasn't the right size for her.” Similarly, Maria stated that they were waiting to “get urgent mod[ification]s on a van to make space for [child], because her head hits the roof of the van.”

### 3.4 The fallibility of the scheme

Multiple participants described issues within the NDIS, with some parents able to adapt their skills to work around such issues. Three subthemes emerged: *inconsistencies within the scheme*; *staff availability and competency varies*; and *fears for the sustainability of the scheme*.

#### 3.4.1 Inconsistencies within the scheme

Inconsistencies within the NDIS were mentioned by participants and noted between the differences in their responses relating to plan values and supports able to be afforded. Linda and Natalie acknowledged good alignment between their child's needs and NDIS plan, which contrasted with their earlier experiences in the scheme. However, Dave and Maria described experiencing challenges that have not been resolved since their child's NDIS acceptance. Dave explained that parents must ask for a plethora of supports, as “you're only going to get … a small percentage of that.” Two participants described insufficient NDIS funds which resulted in privately paying for support workers. Contrastingly, Linda described getting “everything approved” within their child's plan.

Additionally, participants described receiving inconsistent information from NDIA staff. Grace explained that they submitted the “same documentation” twice but had different outcomes.

#### 3.4.2 Staff availability and competency varies

Six participants explained that NDIA staff lacked an understanding of participant experiences raising a child with a complex disability, along with having limited knowledge about disability generally.

I find it really challenging with the NDIS or the NDIA, that I have these people making decisions about my child and about what my child needs, and what my child's like, you know, what my child's reasonable and necessary supports are. They never laid eyes on my child. They don't know him. They don't know who he is. They don't know. (Linda)

Alice recounted experiences trying to get equipment and reported that “the staff don't necessarily know what they are viewing.” Thus, participants expressed the need for more knowledgeable staff with real‐life experiences, so they can “see what we deal with every single day” (Nicole).

Participants also described having positive interactions with NDIA staff and Local Area Coordinators. Alice stated that her Local Area Coordinator “was really onto it, otherwise, there would have been no hope” for equipment that allowed for her child's social participation.

#### 3.4.3 Fears for the sustainability of the scheme

Five participants feared for the sustainability of the NDIS. Linda stated that the misuse of funds reported in the media “really worries me” identifying that it would be “devastating” if the NDIS was unsustainable.

Four participants reported extremely high costs for assistive technology and commented on the hourly rate of therapists under the NDIS.

I think the only real difficulty is, you know, the disability markup that comes on everything. Which, you're like, for example, we really love this beanbag thing, this pod for [child], which was easily movable and would just allow for [child] to chill with us, like if we were like watching TV or chilling out in the living room, or whatever, rather than [child], being in their chair all the time. We really liked this pod, we trialled it, but it was like $6,000 for a beanbag. (Dave)

Grace blamed the NDIS saying they “forced prices up.” Nicole explained that the NDIA decided they wanted to pay therapists “$193 an hour.” She then questioned “why aren't we trusting their judgement,” claiming that therapists' recommendations and reports were often ignored within the scheme.

In relation to the all‐must‐be‐new policy implemented by the NDIA, participants suggested some more cost‐effective alternatives. Two participants mentioned that they would happily purchase second‐hand equipment, as it would allow them to “use that funding elsewhere” (Natalie).

### 3.5 A critical scheme

Participants acknowledged the necessity of the funded services received through NDIS funding to enable daily participation, including during the pandemic. The following subthemes emerged: *vulnerable children during COVID‐19* and *appreciation for the NDIS*.

#### 3.5.1 Vulnerable children during COVID‐19

Multiple participants reported that the NDIA added additional support and carer hours to assist with care and home‐schooling during the pandemic. Alice stated that the NDIA “were actually quite good at putting emergency hours in” which for some participants, allowed for the continuation of employment.

Participants took extra precautions to reduce the risk of their child contracting COVID‐19, as their children were “medically quite at risk” (Grace). Linda noted it was “no one in, no one out,” like Grace who explained that they “cancelled carers” which has “physically taken quite a toll.” Dave stated that during the lockdowns “everyone else kind of got to experience what our life is like, because we're always so restricted in what we can do anyway.”

#### 3.5.2 Appreciation for the NDIS

Six participants stated that without the NDIS, their family “would never be able to provide [child] with what he gets” (Grace). Dave described the NDIS as “an essential service” that is “helping so many families.” Linda stated that “the normality that we've been able to get back into our lives since the NDIS came, you know, into fruition, is amazing.”

Nicole described the challenging nature of the NDIS to access required supports, but compared it to prior experiences:

Now I don't need to do fundraising to get what she needs. Gotta fight for it, you gotta research it, you gotta study it, you've got to work it out and you've gotta find a way, but if you can find the way, it's easier than running a 200 people fundraiser day.

## 4 DISCUSSION

This qualitative study explored the experiences of eight parents of children with cerebral palsy and complex disability, who were very forthcoming about their experiences accessing services for their child through the NDIS for three or more years. Even with NDIS funding for supports and services, children in this study would be unable to participate in daily life without profound levels of unpaid care and parental support provided by parents. It was evident that for parents in this study, access to the NDIS did not replace the need for caregiving or reduce the number of roles and responsibilities that they undertook as parents of a child with a disability. The findings acknowledge the direct care, organising, transporting, advocating and all other tasks (on top of volunteering for the current study) and attests to the demanding role that parents undertake. As partners with parents, and service providers supporting children with cerebral palsy and complex disability, occupational therapists can learn from the experiences described in this study, and contribute to the improvement of services for families.

Children of parents in this study were self‐managed (n = 3), plan managed (n = 3), or both (n = 2). All parents described detailed responsibilities organising, communicating, coordinating and resolving problems in their role advocating for their child with the NDIA. Parents also provided substantial medical care and daily living support, made their own equipment when necessary, transported their child to appointments, and reported conflicting views with the NDIA about what constitutes parental responsibility. Meltzer and Davy (2019) investigated the NDIA policies around supports and noted that the NDIS does not recognise the other roles that families have outside of caring for the participant. Consequently, many parents in this study felt that the NDIA misunderstood the level of caregiving provided and the impacts on their daily life. Parents openly disclosed their mental health issues, inability to work and family breakdowns that had resulted from the demands of their various roles and responsibilities. Thus, the need to access health maintaining and mental health services for parents is a salient finding from the study, and also an area of expertise for occupational therapists.

Individualised funding models (such as the NDIS) require people with disability and their families to have the ability to manage a budget and advocate as necessary (Mazzucchelli et al., 2023). Aligning with the findings in Devine et al. (2022), the attainment of skills in scheme navigation was supported by increased time spent within the scheme. When compared to the previous study with the same participant cohort after only 1 year in the NDIS, parents had described more extreme administrative frustrations, more time delays, and an “impersonal” NDIA (Smethurst et al., 2021). Three years later, parents still described the same challenges, although parents also expressed empowerment over the system and most had achieved substantial supports and services for their child. These findings align with Tracey et al. (2018) that more time within a scheme results in increased skills and competency. Specifically, NDIS participants and their families require support from their inception into the scheme, to maximise use of the scheme, choice, and control. In the current study, parents identified occupational therapists and allied health as assisting their empowerment. Occupational therapists who are knowledgeable about the scheme and expert clinicians can be extremely important to families, thereby demonstrating the need for occupational therapists to stay current and invested in NDIS requirements.

Participants in this study depicted NDIA staff as underprepared for their role within the disability sector. Past research has emphasised the risk to participants when administering staff are not sufficiently capable of delivering a service, which can result in plan inconsistencies and unsatisfied participants (Gilchrist & Perks, 2023). Aligning with previous research, parents in the current study matched the experience of parents who were 12 months into the scheme and viewed the NDIA as lacking empathy and misunderstanding of their daily challenges (Smethurst et al., 2021; Yates et al., 2022).

In the current study, the NDIS was again portrayed as a highly complex and inconsistent scheme, exacerbating stress for parents. Parents noted inconsistencies between the funds received and support acquired through the NDIS, comparable with Malbon et al. (2019) who reported that plan variability was common. Consequently, parents did not feel secure about their child's plan within the NDIS. These findings align with previous research that identified planning reviews and meetings to commonly be a time of increased stress and worry (David et al., 2023). Two parents in the current study were appealing unsatisfactory plans, but six felt satisfied with the supports provided to their own child. Unique to the current study, parents were focussed on threats to the viability of the scheme that supported their child. For example, the high and unsustainable costs for services and assistive technology provoked anxiety in parents. It is well known that children with cerebral palsy have high and expensive equipment needs (Bourke‐Taylor et al., 2014; Tonmukayakula et al., 2018), and it seems reasonable to expect that the NDIS is also aware of this necessity and added responsibility on parents. Many parents suggested that the NDIA review their procedures to include options for purchasing used equipment, as new equipment is preferred and used equipment is disposed of rather than reused.

The COVID‐19 pandemic posed an opportunity for the NDIA to demonstrate the scheme's flexibility and responsiveness to the needs of participants. Zhang and Chand (2023) mapped NDIS funding packages against lockdown durations and found that the average plan value increased over the pandemic. Although more funding was allocated, the NDIS participants in this study were not able to utilise their packages, which further aligned with the 8.85% less NDIS funds spent during the lockdowns in Victoria (Zhang & Chand, 2023). In the current study, parents identified government lockdowns as more impactful on family participation and wellbeing, rather than direct actions or inactions of the NDIA.

Parents in the current study identified the misuse of funding, such as overcharging and dishonesty, as perceived threats to the sustainability of the NDIS. Although only eight parents participated in the current study, their fears may be validated against the reported $6 billion annually being misused within the NDIS attributed to fraud and overcharged services (Dickinson & Yates, 2023). Parents feared resulting funding cuts and thus, strongly desired reassurance from the NDIA that their child's future is protected. Further, parents were concerned about overpricing and waste, such as no provision for using second hand equipment. Occupational therapists are key professionals involved in the provision of equipment, technology, home, and care modifications for this cohort of families. Hence, this affords professionals the opportunity advocate for individual families and for their clients as a whole group with lifetime needs. Occupational therapists are encouraged to advocate for change by reporting the difficulties as well as providing ideas for the solutions to equipment and technology prescription and training. Both the current study and 12 months study, reported instances of injury to children and their parents. The injuries and risks involved in unavailable equipment, unsafe prescription, and use of poorly fitting equipment requires urgent attention to protect the NDIS participant and parent alike.

Allied health professionals accessed via NDIS funds were appreciated by parents in this study for their positive impact on their children and family. Parents predominantly attributed their knowledge of the NDIS to be from allied health professionals. Occupational therapists consider parent education as a major role (Novak & Honan, 2019). Allied health professionals also assisted with attaining assistive equipment; however, the wait times through the NDIS corresponded with the long and emotionally taxing wait times reported by Smethurst et al. (2021). Thus, this issue remains ongoing, and a solution is yet to be devised by the NDIA. Parents also reported to be able to access support workers through the NDIS for which they were grateful. However, parents made it clear that employing support workers did not equate to parental freedom.

Parents undeniably were thankful for the NDIS and held hope for their child and family's future, with hope being an essential element within their lives. Some parents held aspirations that 1 day they would receive sufficient funds to meet their child's needs, whereas other parents wished for the continuation of their child's current NDIS plan. Parents of children with disability often felt an uncertainty about their child's future, particularly about their own ageing and associated capacity to provide care (Shenaar‐Golan, 2017). Thus, all parents in this study aspired for a system that is reassuring, sustainable, and lifelong.

### 4.1 Implications for practice

Allied health professionals and importantly occupational therapists have a key role in providing services that support children, including educating parents about their child's needs, advocating for the child, providing family‐focussed therapy, and the provision of reasonably priced services. Collaborating with parents is important, as empowered parents are more readily equipped to collaborate and problem‐solve within their child's therapy and beyond (D'Arrigo et al., 2020). Occupational therapists have an opportunity to positively impact parental wellbeing and empowerment, through facilitating parental skill development. Thus, family‐centred practice must be implemented, as being responsive to the needs of the whole family has been associated with improved health outcomes for both children with disability and their families (Bailey et al., 2012). Past research suggests that occupational therapists are skilful in navigating NDIS policies and processes and that they can influence the allocations of NDIS funding and supports (Barclay et al., 2020). However, occupational therapists must be able to recognise when families require further support and guidance to avoid overwhelming and disempowering parents (Dodd et al., 2009).

Occupational therapists working within the NDIS have an opportunity to advocate for the wider population of NDIS participants at a legislative level, to evoke a higher level change, and to improve service access, reducing the requirement for families to advocate individually (Stover, 2016). The expertise and holistic perspectives of occupational therapists can help guide the NDIS to ensure responsiveness to the needs of children with complex disability and their families.

### 4.2 Limitations and future research

This study was limited by participants being sampled from one service provider, excluding insights from parents outside of this organisation. However, a homogeneous sample was attained, which allowed for the experiences of the NDIS to be more readily compared, due to similarities in diagnoses, length of time in the NDIS, and access to allied health professionals.

Future research should be directed towards exploring the experiences of parents of children with varying disability to understand a greater range of experiences. The perspectives of allied health professionals working within the NDIS would also be valuable to research to provide insights into providing high‐quality care for children with disability and their families.

## 5 CONCLUSION

In this study, parents had developed a strong reliance on the NDIS to support their child to achieve basic daily care and a more enriched life. However, there were many inconsistencies and ongoing issues that limited choice and control, such as long wait times for assistive technology and unknowledgeable staff. Parents also felt uncertainty about their child's future. Thus, refinement must be ongoing to ensure the NDIS is sustainable and the scheme's purpose is reached to the satisfaction of families. For occupational therapy, the NDIS poses an opportunity to continue to support and advocate for families.

## AUTHOR CONTRIBUTIONS

All authors contributed to this research, including devising the study design and data collection methods. The first author undertook the data collection, with authors one and two contributing to data analysis. The first three authors contributed to editing the overall manuscript. All authors contributed to revising the manuscript prior to the journal submission.

## CONFLICT OF INTEREST STATEMENT

The authors have no conflict of interest to declare.

## Data Availability

Data are not available.
